# Engagement strategies in English and Arabic newspaper editorials

**DOI:** 10.1057/s41599-023-01519-y

**Published:** 2023-01-18

**Authors:** Sharif Alghazo, Khulood Al-Anbar, Marwan Jarrah, Ghaleb Rabab’ah, Mutasim Al-Deaibes

**Affiliations:** 1 The University of Jordan, Amman, Jordan; 2 University of Sharjah, Sharjah, UAE; 3 Khalifa University, Abu Dhabi, UAE

**Keywords:** Language and linguistics, Cultural and media studies

## Abstract

This study explores the use and functions of engagement strategies in English and Arabic newspaper editorials. To this end, the study analyses 80 editorials collected from two popular newspapers (40 from each): *The Guardian* which publishes in English and appears in the UK and *Addustour* which publishes in Arabic and appears in Jordan. Following Paltridge’s (2020) taxonomy, the study utilises a mixed-method approach to assess whether differences in the use of engagement strategies between the two corpora are statistically significant and to identify the functions of the strategies used in the two sets of data. The findings show that there are statistically significant differences between the two languages in the use of some engagement strategies. In particular, Arabic editorials included more reader pronouns and less personal asides than did English ones. In addition, although questioning as an engagement strategy was absent in the Arabic corpus, it was used in the English one to transmit information and circulate knowledge. The findings enrich our understanding of how the editorial genre is constructed, and how editorialists engage with their readers in the two languages.

## Introduction

Media discourse has attracted the attention of researchers who are interested in analysing linguistic and rhetoric aspects of discourse (Alkhalidi and Alghazo, 2022; Rabab’ah et al. 2020; Alzawaydeh and Alghazo, 2018). For example, O’Keeffe and Breen (2007) examined the lexico-grammatical devices which writers of news articles use to express their stance in a large corpus representing over 700 Irish newspaper articles. Al Huneety et al. (2019) analysed lexical cohesion in 105 Arabic newspaper editorials and found semantic relations assist readers in understanding culture-specific expressions in editorials. Bonyadi and Samuel (2013) analysed the textual and rhetorical strategies of headlines in newspaper editorials. Not only is the language of newspapers a source of data for researchers, but also it has long been a resource for teachers to use in the classroom. Baumgardner (1987) examined the pedagogical role of newspapers in the teaching of English grammar and concluded that “the local English-language newspaper affords endless possibilities for use as supplementary content-arid language-focused activities in the ELT [English language teaching] classroom” (p. 250).

The newspaper represents a holistic genre with many sub-genres including news articles, news stories, advertisements, and editorials. In this study, we are interested in analysing the language of the editorial section which plays an essential role in shaping the public view on certain issues of interest. Reynolds (2000) argues that “the newspaper editorial is recognisable as a genre: this is, ethnomethodologically, a member’s recognition, which is an important aspect of the socio-psychological-rhetorical reality of genres” (p. 26). The newspaper editorials play a crucial role in moulding and shaping public opinion. The main objective of the editorial is to present the stance of the newspaper on certain issues and events and to persuade the audience of the view of the newspaper. Biber et al. (2021) argue that newspaper writers—including editorialists—are expected to give a “relatively objective presentation of information … [by] adopting an institutional voice” (p. 9). This necessitates careful crafting of the texts they produce in order not to bring into the discourse any subjective values. In a nutshell, the editorial functions “to comment, via argument mode, on current events, expressed through narrative and description mode” (Reynolds, 2000, p. 27). This implies that careful attention should be paid by the writer of the editorial—who is often the editor of the newspaper—to ensure that the final text fulfils the functions of transmitting the stance of the newspaper and persuading the reader of the taken stance. To achieve these objectives, editorialists make use of a wide range of linguistic and rhetorical strategies to both deliver the intended message and accommodate the reader in the content of the text. Of the many linguistic and rhetorical features upon which editorialists draw is the use of engagement strategies that aim to engage the reader with the text.

Skilled editorialists are cautious about the use of engagement strategies in order to draw the attention of readers and persuade them to subscribe to their and the newspaper’s stance. Indeed, the use of engagement strategies provides a useful account of how editorialists bring their readership into the ongoing discussion of certain issues. Lafuente-Millán (2014) views engagement strategies/markers as “explicit forms of address to the audience which allow writers to invoke the readers and to include them as discourse participants by commenting on their (assumed) knowledge, views or possible reactions” (p. 202). Hyland (2019a) argues that engagement strategies are devices that are used by writers to acknowledge the presence of the audience and fulfil two important functions:

1. The first acknowledges the need to adequately meet readers’ expectations of inclusion and disciplinary solidarity, addressing them as participants in an argument with reader pronouns (*you, your*, inclusive *we*) and interjections (*by the way, you may notice*).
2. The second purpose involves rhetorically positioning the audience, pulling readers into the discourse at critical points, predicting possible objections and guiding them to particular interpretations. These functions are mainly performed by questions, directives (mainly imperatives such as *see*, *note* and *consider* and obligation models such as *should*, *must*, *have to*, etc.) and references to shared knowledge. (p. 63, italics in original)

Therefore, writers—including editorialists—write to express and impress by carefully attending to their readership so as to express their views and convince readers to buy into the position of the writer and/or the newspaper, in the case of editorialists. Lafuente-Millán (2014) contends that in order for writers to do so, they “need to project a competent disciplinary identity and to take into account the preferred discursive patterns of their readership” (p. 202). Hyland (2019a, p. 120) also emphasises the fact that “engagement markers explicitly address readers to selectively focus their attention or to include them in the text.” Although there are some studies that examine the use and functions of engagement strategies in various types of texts (e.g., McGrath and Kuteeva, 2012; Sayah and Hashemi, 2014; Paltridge, 2020), engagement as opposed to other interactional markers such as stance and voice “has too long slinked around in the low rent areas of discourse analysis” Hyland (2019b, p. XI). More so are contrastive analyses of the functions of engagement strategies in the newspaper editorials written in two distinct languages (e.g., English & Arabic). The present comparative study is a functional analysis^1^ of how editorialists use engagement strategies to accommodate their audience in both English and Arabic. In particular, the study seeks to answer two main research questions:

1. How do newspaper editorialists connect with their readers in English and Arabic?
2. What are the similarities and/or differences (if any) in the use of engagement strategies between English and Arabic newspaper editorials?

## Theoretical framework

Editorialists, and all competent writers for that matter (see Hyland (2001) for a similar argument about academic writers), pay considerable attention to three elements: the writer, the text, and the reader. Hyland (2019a) elaborates this by arguing that any text (written or spoken) “includes expressions which refer to the text producer, the *imagined receiver* and the evolving text itself” (p. 16, italics added). It is essential to clarify the use of the word ‘imagined’ in the quote. Hyland (2019a) shows that the notion of audience is “notoriously elusive” (p. 13) and that analysts have different views on what constitutes the audience. Some, he continues to note, believe that “audience is real people outside a text whom the writer must consider and accommodate,” and others see it as “a fiction embodied in the writer’s rhetorical choices” (p. 13). Therefore, writers imagine the interests of readers and “construct an audience” because in many cases we do not know “who we are addressing,” or there might be “multiple audiences” (Hyland, 2019a, pp. 13–14). Despite this, all text producers (writers or speakers) attend to their imagined readers and attempt to construct their text in collaboration with that imagined audience. This is how writing becomes interactive and more persuasive. Zainuddin and Moore (2003) found that successful writers pay attention to the concerns of their audience which, in turn, affects the quality of their texts.

Engagement strategies are essential rhetorical tools for writers to guide readers through the text. Curry and Stroud (2021) argue that reader engagement is an intriguing facet of academic and professional texts because it “is concerned with how readers are included, exploited, and positioned in texts, using … markers like reader pronouns and questions” (p. 1). In mainstream literature, there appeared some frameworks that provide perspectives on how to analyse and classify engagement strategies/markers. Some of these have drawn upon Halliday’s (1994) Systemic Functional theory of language which stipulates that texts should be examined holistically to fulfil three metafunctions: ideational, textual, and interpersonal. While the theory of Systemic Functional Linguistics is a valuable ground for the analysis of text, it is more linguistically grounded than metadiscourse theories which stress that “metadiscourse cannot be regarded as a strictly linguistic phenomenon at all, but must be seen as a rhetorical and pragmatic one” (Hyland, 2019a, p. 29). This study, as noted above, draws on Paltridge’s (2020) taxonomy which is based on previous taxonomies (Hyland, 2001, 2005; Hyland and Jiang, 2016). Table 1 lays out the list of engagement strategies, their realisations, and examples of each.

**Table 1:** Engagement strategies, realisations, and examples (Paltridge, 2020, pp. 10–11).

Engagement strategy	Realisations	Examples
*Directives*, instructions to the reader which direct readers (a) to another part of the text or to another text, (b) how to carry out some action in the real-world, or (c) how to interpret an argument.	Imperatives (e.g. *note that, consider, refer to, see*)	*Rephrase* the first sentence. *See* below for a few examples.
	Modals of obligation (e.g. *should, must, ought*)	The authors *should* refer to the difference between indicative and informative abstracts You *must* add specific examples of how language learning and creativity are related in order to make your argument.
	Adjectival predicate expressing judgements of importance/necessity controlling a complement *to*-clause (e.g. It is essential *to*, It is necessary *to*)	*It is essential to* reference Halliday here.
*Questions*, inviting direct collusion because they address the reader as someone with an interest in the issue the question raises and the good sense to follow the writer’s response to it, often rhetorical, presenting an opinion as an interrogative.	Interrogatives	Are you going to provide a technical definition? Should ‘genre’ read ‘discipline’? Is there a way you can vary the phrasing?
*Reader pronouns*, bringing readers into the discourse, normally through second person pronouns, particularly inclusive we which identifies the reader as someone who shares similar ways of seeing to the writer, claiming solidarity, acknowledging the presence of the reader.	*You, your, we, our, us*	The approach *you* have taken would be of interest to many ESP practitioners This has ramifications for *your* recommendation *We* (as applied linguists) are forced to decontextualise *our* examples
*Personal asides*, briefly interrupting the argument to offer a comment on what has been said, adding more to the writer-reader relationship than to propositional development.	e.g. bracketed text, use of –,	(this language group is undefined) In the abstract you say ‘seven years ago’ – why not say ‘in 1997’?
*Appeals to shared knowledge*, explicit signals which ask readers to recognise something as familiar or accepted	e.g. *As we know, obviously, naturally, of course*	*As we know*, writing in academe serves two main functions: learning and display. Such articles are *naturally* of real salience and interest to academics, including this reviewer. *Of course*, you will be discussing this context, but you need to demonstrate, first, that the issue you and your students face is common in ESP contexts throughout the world.

## Literature review

As noted earlier, engagement as an interactional metadiscourse feature has not yet received due attention in metadiscourse research, compared to other features of stance and voice (Alghazo et al., 2021). Engagement has often been examined in a subsidiary manner to stance (see Hyland, 2005; Alghazo et al., 2021; Herzuah, 2018; Abusalim et al., 2022). Hyland (2019b) argues that engagement “has always seemed the poor relation in discussions of interaction” (p. XI) although it is an essential feature of interaction. In fact, as writers and speakers, we need to acknowledge our readers and listeners and make our discourse more interactional by means of engagement strategies/markers. As Jiang and Ma (2019) put it, we “must employ recognised ways to express arguments and initiate social engagement, which readers find familiar, appealing and persuasive” (p. 32). Despite the paucity of research on engagement for a long time, the last decade produced a number of studies and reference materials on engagement. A notable example is an edited volume by Guinda (2019) which includes several studies on the use of engagement strategies in professional discourse. In addition, there now exists a number of empirical and corpus-based analyses of engagement strategies in various types and genres of discourse. In what follows, we focus on studies that explicitly examined engagement in various genres.

Of the most recent explorations of engagement is a study by Paltridge (2020) who examines the use and functions of engagement strategies in reviewers’ reports on submissions to journals by analysing 97 reports totalling 71,661 words. Following existing taxonomies of engagement, the researcher identified the strategies reviewers use in their communication with authors. The results indicate that the strategy of directives was the most commonly used one, often used indirectly or hedged. The findings also show that there was the frequent use of reader pronouns as an interpersonal strategy that reviewers use to engage with authors. Most notable is the researcher’s conclusion that the engagement strategies used by reviewers are not genre-specific but “reflective of the discourse community’s expectations for interactions within the community as a whole” (p. 21).

Hyland and Zou (2021) looked into the use and functions of engagement strategies in 3 min Thesis (3MT) presentations in which doctoral students present their work to an audience in only 3 min and using one slide. The researchers analysed a corpus of 120 3MT presentations from both hard and soft sciences to see how doctoral students relate to the audience and accommodate them in an “artificially controlled, competitive environment” (p. 21), particularly that the audience in this genre is a non-specialist one. The results indicate that the strategy of hearer mention was the most frequently employed in both corpora. Moreover, the study found that the strategy of directives was more frequent in the hard science presentations and that questions were used equally in the two corpora.

In a corpus-based contrastive investigation of the use of engagement strategies/markers, He and Abdul Rahim (2019) conducted a comparative analysis of engagement markers in economics research articles and opinion pieces using AntConc to elicit data for analysis. Hyland’s (2005) was used to identify the engagement strategies used in both corpora. The findings revealed that there were more engagement markers in the research articles than that in the opinion pieces. In addition, the study found that the strategy of directives was the most frequently used by writers in the two genres. The results also indicated that the least frequently used engagement markers were the questions and personal asides. The study provided implications for English for specific purposes (ESP) studies and second language pedagogy.

Lafuente-Millán (2014) examined—through corpus analysis—the connection between the use of engagement strategies and three variables: the language of publication, the context of publication (local vs. international), and culture by adopting a contrastive analysis of two sets of data collected from English and Spanish research articles in business management. The results of the quantitative analysis revealed that the three variables have an impact on the use of engagement strategies in the examined form of discourse. In particular, the findings indicated that “national culture is … [a] more powerful factor within the multivariate equation which regulates the use of … [engagement] strategies” (p. 219). In addition, the study found that, in the Spanish corpus, there appeared to be a transfer of certain rhetorical patterns from the L1 into the L2 and that proficiency in the L2 might have impacted the use of certain engagement strategies by the Spanish researchers.

Yang (2021) investigated the use of engagement strategies across participants by contrastively analysing two corpora of letters of advice constructed by two types of writers: governments and hospitals addressing two types of readers (citizens and staff, respectively). The study analysed data collected from Chinese letters of advice (*N* = 120) produced by governments and hospitals during the COVID-19 pandemic. The results showed that both sets of data included engagement markers and that their use did not significantly vary based on the issuing agencies. In particular, the findings showcased that there were no differences in the use of engagement strategies by the two agencies and that “there … [was] little change in the use of engagement markers as the reader’s role shifts from staff to citizen” (p. 187).

Based on the foregoing, we notice that studies which contrastively explore the use and functions of engagement strategies/markers are scarce, and more so are studies that investigate engagement in Arabic and English editorialists. Thus, this study aims at comparing journalistic writing practices in two different languages (English and Arabic) by examining the use and functions of engagement strategies. Editorials are chosen in this research because of their subjectivity and explicitness in representing the newspaper’s position which differentiates them from academic prose (Hyland, 2019a).

## Methods and procedures

The data were collected from the editorial section of two popular newspapers representing the two languages compared in the study: *The Guardian* which publishes in English and appears in the UK and *Addustour* which publishes in Arabic and appears in Jordan. A corpus of 80 editorials (40 from each newspaper) was constructed for analysis. The editorials were retrieved from the website of the two newspapers. The editorials which were published between 2020 and 2021 were selected for reasons of recency so that the conclusions describe the genre at hand more accurately. Each set of editorials was transferred into a Microsoft Word file for analysis. In the analysis phase, a mixed-method approach, utilising quantitative and qualitative measures, was adopted. The analysis is based on Paltridge’s (2020) taxonomy^2^ which is adapted from Hyland (2001, 2005) and Hyland and Jiang (2016). First, a list of potential engagement markers in both English and Arabic was prepared by reviewing previous studies in the literature (e.g., Crismore and Farnsworth, 1990; Hyland, 1998, 2001, 2005; Abdollahzadeh, 2011; Taki and Jafarpour, 2012; Hyland and Jiang, 2016; Alotaibi, 2018). Statistical analysis tests were used to find similarities and/or differences in the use of engagement strategies in the two sets of data. In particular, the Mann–Whitney *U* test was run to count the frequencies and percentages of engagement strategies in both corpora.

In order to identify the functions of the engagement strategies/markers in each set of data, a functional analysis was conducted. In doing so, we follow Hyland (2019a) who argues that an essential step in the analysis of engagement strategies is to emphasise “meanings in context” and to show “how language is used, not what a dictionary says about it” (p. 28). We examined each marker within its context to make sure that the marker is considered to be an engagement strategy. Each engagement feature was highlighted and then categorised based on the five types of engagement strategies in the taxonomy. For a fine presentation of the analysis, the Arabic examples were translated carefully into English after ensuring that they were considered engagement strategies. The analysis was then validated by three experts in discourse studies.

### Findings

The data analysis included a comparison between the two sets of data which led to answering the research questions of this study. Three forms of calculations were conducted: the frequencies, percentages, and frequencies per 1000 words. The number of occurrences of each type of engagement strategy was calculated to identify the similarities and/or differences between the two sets of editorials. In accounting for frequencies per 1000 words, we follow the convention used in metadiscourse and engagement studies (see Hyland, 1998, 1999; Noorian and Biria, 2010; Fu and Hyland, 2014; Liu and Zhang, 2021; Wu and Paltridge, 2021) which allows researchers to present more accurate comparisons in terms of metadiscourse use. Table 2 shows the frequencies, percentages, and frequencies per 1000 words of engagement strategies in the two corpora.

**Table 2:** Frequencies, percentages, and frequencies per 1000 words of the two corpora.

*Strategy*	The Guardian			Addustour		
	Freq.	%	% per 1000 words	Freq.	%	% per 1000 words
Reader pronouns	49	20.4	1.99	219	94	16.43
Personal asides	116	48.3	4.70	3	1.3	0.23
Appeals to shared knowledge	5	2.1	0.20	5	2.1	0.38
Directives	49	20.4	1.99	6	2.6	0.45
Questions	21	8.8	0.85	0	0	0.00
Total	240	100	9.73	233	100	17.48

As shown in Table 2, 240 engagement markers were found in the English corpus, with all types of engagement strategies in use. The most frequent engagement strategy was *personal asides*, which comprised 48.3% of the strategies; this is followed by *reader pronouns* (20.4%) and *directives* (20.4%). There were also *questions* in the English editorials, with 8.8% of the strategies. However, no questions were found in the Arabic set of editorials. The least frequent engagement strategy in the English set was *appeals to shared knowledge*, with 2.1% which was exactly the same as that of the Arabic editorials (2.1%). In the Arabic set of editorials, 233 engagement markers were used with the absence of questions from this set of editorials, as just mentioned. The most frequent strategy in the Arabic editorials was *reader pronouns*, with 94% of the strategies. Table 2 also shows that the second most frequent strategy in the Arabic corpus was *directives*, with 2.6%; this was followed by *appeals to shared knowledge*, with 2.1% and *personal asides*, with 1.3%.

As noted earlier, the Mann–Whitney *U* statistical analysis test was used to measure the differences and/or similarities between the two groups of data. The Mann–Whitney *U* test compares two independent groups to determine if there are differences in medians between them. The results of the Mann–Whitney *U* test, as shown in Table 3, revealed that not all engagement strategies were present in both sets of editorials. However, there were significant differences between the two groups in the overall use of engagement strategies. The Mann–Whitney *U* test results also showed a significant difference between English and Arabic editorials in the use of *reader pronouns*. The results also revealed that the use of *personal asides* was similar in the two sets of editorials but showed significant differences between the two groups of editorials among the rest of the engagement strategies (*appeals to shared knowledge, directives*, and *questions*).

**Table 3:** The Mann-Whitney U test results.

Strategy	Corpus	Mean Rank	Sum of Ranks	Mann–Whitney *U*	*Z* Value	Sig
Reader Pronouns	Arabic	46.68	1867.00	553.000	−2.488	0.013*
	English	34.33	1373.00			
Personal Asides	Arabic	40.49	1619.50	799.500	−0.013	0.990
	English	40.51	1620.50			
Appeals to Shared Knowledge	Arabic	38.50	1540.00	720.000	−2.039	0.041*
	English	42.50	1700.00			
Directives	Arabic	37.04	1481.50	661.500	−2.430	0.015*
	English	43.96	1758.50			
Questions	Arabic	33.50	1340.00	520.000	−4.069	0.000*
	English	47.50	1900.00			
*Total Engagement Strategies*	Arabic	58.25	2330.00	90.000	−6.915	0.000*
	English	22.75	910.00			

*Refers to a statistically significant difference between the two sets, the mean difference is significant at the level (0.05) (*a* = 0.05).

Now that we have presented the results quantitatively, we turn to the qualitative analysis of the data obtained from the two corpora. We present our analysis for each engagement strategy with illustrative examples to explicate their functional use in discourse.

### Reader pronouns

The use of reader pronouns refers to the way personal pronouns such as the inclusive *we* and the second-person pronoun *you* in interacting and communicating with readers. Writers make use of reader pronouns as an engagement strategy to involve the reader and to make the text more compelling and persuasive (Scollon et al., 2012). As shown above, in the section, the use of reader pronouns in the analysed Arabic editorials (94%) was greater than the use of reader pronouns in the English ones (20.4%). According to the results of the Mann–Whitney *U* test, a significant difference was detected between the two languages in the usage of reader pronouns within the editorial section. In particular, the use of inclusive *we* were more prominent and greater than any other type of personal pronoun in the editorials of both languages (26 times in the English editorials and 147 times in the Arabic ones). This might be attributed to the fact that “inclusive pronouns can act as positive politeness devices by describing and/or critiquing common disciplinary practices and elaborating arguments on behalf of the community” (Harwood, 2005, p. 343). We may interpret this finding by referring to the Arabic culture which is said to show a collectivist identity in different discourses. This interpretation is inspired by Na’s and Choi’s (2009) argument about the connection between culture and the use of first-person pronouns. They found that there is a difference between Koreans and Americans in the use of first-person plural pronouns and attributed the high frequency in the use of first-person plural pronouns by Koreans to the fact that Koreans have “a more collectivistic orientation” than Americans do and that “first-person plural pronouns are more readily available to collectivists than to individualists” (p. 6). Some examples of the use of reader pronouns in both corpora follow:

1. And that is the best *we* can hope for over the next three decades. (“The Observer view on the urgency of”, https://www.theguardian.com/international).
2. *We* now have fewer than 100 days before the United Nations’s Cop26 climate change conference opens in Glasgow, when world leaders will be given one last, clear chance to limit climatic mayhem. (“The Observer view on the urgency of”, https://www.theguardian.com/international).
3. بالمحصلة *نحن* أمام مرحلة جديدة واعدة مليئ بالمشاريع الحيوية، وهي بالمجمل حصيلة رؤى ملكية هدفت إلى جعل العقبة مقصدا سياحيا واستثماريا وتجاريا، وبوابة بحرية إقليمية، تنعكس آثارها على معدلات النمو وخلق فرص العمل والتخفيف من أرقام البطالة. (“الملك في العقبة“، الدستور، ٢٠٢١).
  As a result, *we* are in front of a new promising stage which is full of vital projects. This stage is the outcome of the royal vision that aims at making Aqaba a tourist, economic and commercial destination as the regional coastal gateway. This new phase will impact the rates of growth and unemployment and it will create new job opportunities. (“The King is in Aqaba”, https://www.addustour.com/).
4. شباب*نا* قادرون على إحداث التغيير الإيجابي عبر مشاريعهم. (“شبابنا قادرون على“، الدستور، ٢٠٢١).

*Our* youth can create a positive change through their projects. (“Our youth are capable”, https://www.addustour.com/).

The above-mentioned examples illustrate how reader pronouns can be used as a strategy to engage the reader in the discussion, especially by the use of the plural pronoun *we* in English and the plural independent personal pronoun in Arabic نحنُ. In the three first examples, the editorialists used the pronoun *we* to express a view that is shared by the audience and to persuade them to accept it. The fourth example shows how the plural suffixal pronoun /naa/ ــــــَنا which means (our) can be used to address the reader as part of the dialogue created in the text.

### Personal asides

Personal asides are defined as comments made by the writer within an argument to direct the reader to another related idea; they form an abrupt stoppage to engage the reader into continuing the argument. Hyland and Jiang (2016) referred to personal asides as an intimating sharedness and stated that “it is an intervention simply to connect, to show that they are all, writer and readers alike, engaged in the same pursuit and can draw on shared understandings, if not of actual content, then at least of what might be considered a relevant aside” (p. 18). In this study, the results of the Mann–Whitney *U* test showed no significant differences in the usage of personal asides between English and Arabic editorialists. The following example shows the use of personal asides in both sets of data:

5. For the sake of the Lebanese people, and out of obvious self-interest, the international community—*and that includes too-silent Britain*—must take the lead. (“The Observer view on the unfolding crisis”, https://www.theguardian.com/international).
6. When the government tells people that they can do something—*and when ministers say that they will do something*—many people understandably interpret that as meaning that it is safe to do something. (“The Guardian view on relaxing Covid”, https://www.theguardian.com/international).
7. ليجسد هذا التواصل مدى شعور القائد بأبناء شعبه*، وهو ما يدفعنا جميعاً لتعظيم هذه القيم النبيلة،* وهي رسالة تدلل أيضاً على مدى اهتمام جلالة الملك بسير تطور عمل المركز رغم عديد التحديات (“طوبى لكافل اليتيم“، الدستور، ٢٠٢١).
  This communication reflects how much the leader feels about his people*, which motivates us all to maximise these noble values*, a message that also demonstrates how much His Majesty the King is interested in the evolution of the Centre’s work despite the many challenges.
8. نحتاج اليوم كما يؤكد جلالة الملك عبد الله الثاني إلى إحياء الروح التي بني بها وعليها الأردن*، فلا مكان لليأس بيننا،* وقيادتنا وشعبنا لا يعرفون المستحيل (“مئوية الدولة“، الدستور، ٢٠٢١).

Today we need to revive the spirit upon which Jordan was built as His Majesty King Abdullah II asserts*, there is no place for despair between us,* our leadership and our people do not know the impossible.

Examples (5)–(8) illustrate the use of personal asides in English and Arabic editorials. The editorialists openly made a comment on the issues under discussion using parentheses and commas as in the abovementioned examples. In the case of editorials, personal asides are not quite personal. Since editorials represent the institution’s viewpoint rather than the writer’s personal viewpoint, personal asides are among the most explicit ways of offering this standpoint. It can be noticed from Examples (5) and (6) that the attention is drawn to the writer’s comment through the use of personal asides. Khabbazi Oskouei (2011, p. 128) claimed that “asides are a temporary departure from the main topic. They are, arguably, used by the writers to convey a special message specifically directed at the readers and are used to establish a special relationship with them”. And this is what the editorialists exactly did in Examples (7) and (8): They made a special statement to the readers and galvanised them into action.

### Appeals to shared knowledge

Appeals to shared knowledge refer to a strategy that writers use to engage readers by stating a piece of information that is shared between the writer and reader. In simple words, the writer attempts to treat the reader as a fellow member by using appeals to shared knowledge (Hyland, 2005). A significant statistical difference between the two languages was evident in the use of appeals to shared knowledge. Below are some examples of appeals to shared knowledge from the English and Arabic set of editorials.

9. *Needless to say*, it is children and women who suffer most from the missing provision, especially those from poorer backgrounds (“The Guardian view on early years”, https://www.theguardian.com/international).
10. Though often hit by the long-term shift to online retail—which the pandemic, *of course*, accelerated—such places continue to knit the social fabric together in vital ways. (“The Guardian view on the future of”, 2021).
11. هذه الرسائل الملكية الهادفة إلى توحيد الصف العربي، تصب *بطبيعة الحال* في إعادة البوصلة وحشد الجهود الداعمة لعدالة القضية لفلسطينية، حيث ضرورة التوصل إلى حل عادل وشامل لها على أساس حل الدولتين، ووفقا لمبادرة السلام العربية، وقرارات الشرعية الدولية. (“حرص أردني على التضامن العربي“، الدستور، ٢٠٢١)
  These royal messages aimed at unifying the Arab class *naturally* serve to restore the balance and mobilise efforts in support of the justice of the Palestinian cause. It is also necessary to achieve a just and comprehensive solution based on the two-state solution, in accordance with the Arab Peace Initiative and the resolutions of international legitimacy.
12. لم يعد مقبولا السكوت عن جرائم المحتل، وصاحب الأرض والحق والقضية سينتصر *حتماً*. (“ سـنـبـقـى متسلحين بتضحياتنا“، الدستور، ٢٠٢١)

Silence upon the occupier’s crimes is no longer acceptable. And the owner of the right and the land will *inevitably* triumph and prevail.

The reader should not be infuriated by the flippancy or lack of seriousness in addressing him/her within the editorial, and the best method to overcome this point as an editorialist is to use appeals of shared knowledge. In Examples (9) and (10), the editorialist referred to the common ground with the reader by using some multi-word expressions such as ‘needless to say’ and ‘of course’. Examples in (11) and (12) show how single-word expressions (e.g., “حتماً”, ‘inevitably’) and multi-word expressions (e.g., “بطبيعة الحال”, ‘of course’) can be used as appeals to shared knowledge in editorials. Hyland (2001) stated that “readers can always refute claims, and this gives them an active and constitutive role in how writers construct such claims” (p. 549). Therefore, editorialists of the two languages referred to these appeals of shared knowledge to promote the active involvement of their readers.

### Directives

Directives are used to guide the reader and advise on how to run an action in the real world. It should be mentioned that, generally, directives are used to express the need to take a move and perform a certain kind of action using some modal verbs (e.g., *should* and *must*). A significant statistical difference was found between the two languages in the use of directives. Directives were less in Arabic editorials (2.6%), compared to their English counterparts (20.4%). This finding is consistent with that of Al-Rickaby (2020) who compared stance and engagement markers in English and Arabic newspaper opinion articles. Al-Rickaby (2020, p. 192) reported that “[D]irectives, however, are more used in the English corpus than in the Arabic corpus”. This can be attributed to what Wilson et al. (1991) mentioned in their analysis of directives as they argued that “directives constrain the target’s autonomy since by definition this class of acts is used to elicit behaviours which the target otherwise would not have performed” (p. 217). Perhaps, Arabic editorialists attempt to avoid any face-threatening acts toward the readers by minimalizing the use of directives. This view that draws on Brown’s and Levinson’s (1978) Politeness Theory provides an embedded cultural prospect of how the use of directives will affect the reader’s acceptance of the utterance. Editorialists’ awareness of cultural values will always impact their linguistic choices, especially their choice of using directives to directly send a message to their target audience. Given that editorials have a persuasive and argumentative nature, the usage of directives can be quite sensitive because of the possibility of threatening the readers’ faces (the positive and negative). This idea can be a possible interpretation for the lower frequency of directives in Arabic editorials. The following are some examples of the use of directives in both sets of data.

13. We *should note*, too, that Apple’s new feature is called Focus—a nod to cancelling distractions and working harder. (“The Guardian view on work-life balance”, https://www.theguardian.com/international).
14. Today, as in the past, responsibility *should be* widely shared. (“The Guardian view on the climate”, https://www.theguardian.com/international).
15. وهو منجز متراكم وإرث *يستوجب منا* مزيداً من العمل والإنجاز. (“الإصلاح الإداري مفتاح“، الدستور، ٢٠٢١).
  This is a cumulative achievement and legacy that *must be* followed by more work and achievement.
16. إن الجولة الملكية في الجنوب، تدفع كل مسؤول إلى الاقتداء بنهج القائد، فالأردن بلد خير وعطاء، وسواعد أبنائه وطاقاتهم لا تحتاج سوى إلى التبني والإسناد، *وعلى الجميع اليوم* في القطاعين العام والخاص مسؤولية مشتركة. (“بالعزيمة والإصرار تنهض“، الدستور، ٢٠٢٠).

The royal tour of southern Jordan prompts every person in charge to follow the steps and approach of His Majesty the King. Jordan is a good and giving country with the power and strength of its youth, and Jordan needs nothing but support and help. Today, we all *must have* the same shared responsibility in the public and private sectors.

In the first two examples from the English corpus, the modal verb *should* be mainly used as a directive to engage readers in the text. In general, the function of the modal verb *should* in English is to give advice, make recommendations or talk about obligation. As can be noticed, *should* be used as a directive to interact with the readers and to involve them in the discussion. Examples (15) and (16) from the Arabic set of editorials illustrate that modals of obligation can be used to reinforce the persuasive power of the editorial. The use of *must* shows the strong desire that the writer has to involve, engage, and guide the readers.

### Questions

Questions or interrogative statements are classified as a type of engagement strategy that is used to give a sense of emotional involvement to the reader. Biber et al. (2021, p. 207) argued that “by choosing an interrogative form, the speaker appears to let the addressee be the judge, but no overt response is expected”. A significant difference was found between the editorials of the two languages in the use of questions. While questions were absent in the Arabic editorials, questions existed in the English ones amounting to 8.8% of the total engagement strategies. This can also be connected to Sperber’s and Wilson’s (1997) notion of relevance which clearly highlights the systematic way of connecting and communicating with humans. Sperber and Wilson (1997, p. 145) argued that “the pursuit of relevance is a constant factor in human mental life, and that it is systematically exploited in human interaction”. A possible explanation for the absence of questions in Arabic editorials is perhaps the teaching of journalistic writing etiquette in Arabic. Journalists might be told that avoiding questions in opinion articles will protect the reader’s personal space and privacy.

17. In 2011 a repressive, authoritarian government collapsed because it proved unable to meet people’s demands. *Why would its return solve anything?* (“The Guardian view on Tunisia”, https://www.theguardian.com/international)
18. *Can we learn to appreciate our own old clothes as well as other people’s?* (“The Guardian view on secondhand clothes”, https://www.theguardian.com/international)
19. *Will the BBC successfully continue to prove that it is a valuable anchor to British society, providing trusted news, information and entertainment of the highest quality?* (“The Guardian view on the BBC”, https://www.theguardian.com/international)
20. And even if they take off, will options like these be like parental leave for fathers (too often scuppered by fear of censure), or “sleep hygiene”—which in practice can separate those who have a choice from those (with childcare duties, multiple low-wage jobs) *who do not?* (“The Guardian view on work–life balance”, https://www.theguardian.com/international).

In (17), the writer used a wh-question to highlight a point for further reflection in the future. The editorialist in the following two examples used a yes/no type of question. In yes/no questions, “the addressee is expected to supply a truth value by answering yes or no” (Biber et al., 2021, p. 208). In the last example, an opinion is given to foster dialogue through the use of a rhetorical question.

## Discussion

This paper has investigated the engagement strategies employed in English and Arabic editorials collected from two newspapers in the UK and Jordan. Some statistically significant differences were found between the two languages in the use of some types of engagement strategies (e.g., reader pronouns, appeals to shared knowledge, directives, and questions). The data analysed were then presented to understand the nature of engagement strategies in the editorial genre of the two languages (English & Arabic). Although Arabic editorialists in *Addustour* newspaper deployed reader pronouns more frequently, questions were absent from their editorials. It is difficult to explain this result, but it might be that Arabic editorialists are trying to address readers implicitly to avoid being offensive and impolite. The research has also shown that English editorialists utilised more personal asides, compared to their Arabic counterparts. Perhaps, the high frequency of personal asides in English editorials may partly be explained by the genre of editorials and opinion pieces like that. Therefore, it seems possible that the high rate of personal asides in English editorials might be related to the representation of the newspaper’s opinion and voice in the editorial section. It is also to be noted that no significant difference was found between the two sets in the use of personal asides. Personal asides were present in Arabic editorials, but with less frequency (1.3%). In addition, editorialists in *the Guardian* intended to use questioning as a way of transmitting information or circulating knowledge. It was evident that editorialists in the two languages were concerned with the establishment of relationships with readers by means of engagement markers. Consistent with the literature, appeals to shared knowledge and directives are used to address the public and to have a broader audience. Editorialists attract the attention of their readers and get a larger readership thru direct communication or engagement with readers.

Indeed, journalistic texts among other forms of written language lend special significance to the audience. The journalistic genre focuses on the persuasion of the reader with the arguments put forward by the writer. The rhetorical devices and engagement strategies can be considered among these methods through which the writer can represent and convince the audience of his/her viewpoint in a journalistic text. In specific, editorials were designed to promote certain ideas and ideologies to the audience which makes these editorials revolve around the persuasion of the audience. Given that persuasion takes place in the editorial section of the newspaper, the analysis of newspaper editorials would help in identifying the linguistic features utilised in the persuasion of the audience from different cultures and language backgrounds. In fact, one of the linguistic features that can be utilised to establish a writer–reader relationship is engagement markers. Hyland and Jiang (2016, p. 29) described engagement by stating that: “engagement thus involves connecting texts with readers and with disciplinary cultures”. Engagement is especially important in editorials because of the wide and respectable readership of these texts. Journalistic texts as a form of written interaction involve the anticipation of the responses of the potential or imagined reader (Martin and White, 2005, p. 92). Therefore, the writer is always concerned with the methods of convincing the target audience from various linguistic and cultural backgrounds. And here comes the significance of engagement markers as “the devices that explicitly address readers, either to focus their attention or to include them as discourse participants” (Hyland, 2019a, p. 63).

Several factors are found to influence the use of engagement strategies and the ways of persuading the reader with a certain standpoint such as the cultural background and the native language of the writer and the reader. A wide range of studies discussed the idea that cultural background plays an important role in the process of writing and the perception of written texts, and this gave rise to what is known as “intercultural rhetoric”. The term “intercultural rhetoric” can be broadly defined as the influence of one’s first language on the acquisition of second or foreign language writing. The usage of certain linguistic features like, engagement markers depends upon users’ first languages or cultures. Another point to be mentioned here is that the contrastive analysis of editorials relates to the theory of linguistic relativity defined by Connor as “the notion that patterns of language and writing are culture-specific” (p.1). Consequently, this study is a chance to reflect on culture-specific rhetorical strategies that influence the language of newspaper editorials. This study is significant because of its implications for language learning and teaching. Connor and Traversa write:

Teachers of writing for specific disciplines can benefit from the comparison of corpora of discipline-specific texts in different languages in order to identify potential pitfalls for their students. Such corpora comparisons thus help teachers to understand reasons for potential mismatches in the formulation of specific text types by students. (p. 19)

## Conclusion

The present study contributes to the understanding of how engagement strategies can be used in persuasive and argumentative texts. In fact, engagement strategies in opinion pieces can reflect how the writer is trying to be considerate of the reader’s needs. What makes this research significant is that it compares the use of engagement strategies by two groups of writers from two different linguistic and cultural backgrounds. The contrastive analysis of the English and Arabic editorials adds to the knowledge about intercultural rhetoric. The findings of this study are of interest to teachers of professional and journalistic writing in both languages. Writing workshops for novice journalists might draw on these findings to raise awareness about engagement strategies in persuasive writing. Language training classes that equip journalists for the demands of a fast-changing multimedia industry should emphasize the role of engagement strategies in opinion articles. Instances and applications of engagement strategies in English and Arabic ought to be included in journalism diplomas and qualifications of the two languages.

This paper contributes to the overall understanding of the concept of engagement in specialist kinds of texts (i.e., editorials). The comparison between the two languages adds more to the perception of the role of engagement strategies in argumentative writing. The findings reported here have an impact on the teaching of “writing for professionals” courses, in which they could incorporate such engagement strategies. Engagement strategies as tools for addressing the reader’s needs and expectations would help in achieving the desired outcome from editorials efficiently and effectively. These questions would help teachers of journalistic writing in both languages in training students and early career journalists to meet the expectations of their readers. The effective use of engagement markers in any language makes the editorial easier to understand and more attractive. If the editorialist is aware of the functions of engagement strategies and can use them efficiently, this would make the editorial section available to a broader readership. Carroll (1968, p. 113) argued that the contrastive analysis of texts helps teachers to predict the possible interferences from “native language habits”.

This study has pedagogical implications for the teaching of journalistic writing in both languages where early career journalists need to be trained on the usage of these engagement strategies. The findings of this study have a number of practical implications. In terms of material design, this study suggests the integration of engagement strategies in journalistic writing courses in both languages. The explication of engagement strategies to students of journalistic writing would help in raising awareness of their significance in persuasive and argumentative texts. Awareness of engagement markers’ use would help learners of the two languages (English & Arabic) in enhancing their comprehension of the public discourse in such languages as well. Another implication of this study is that students of languages for specific purposes (e.g., English for journalism courses and Arabic for media and journalism courses) should be taught about engagement strategies as rhetorical devices used in building and supporting arguments. Richardson (2013, p. 2) pointed out that “journalistic discourses are always socially situated, therefore analysing them requires more than a list of text-linguistic concepts”. In this study, the analysis of editorials provided some clues about how editorialists in English and Arabic used engagement strategies to socially interact with their audiences. This understanding of how editorialists employed engagement strategies has an impact on the teaching of linguistic argumentation in the field of journalism. Training journalists and editorialists on how to deploy these engagement strategies in their texts will affect their writing practices. For instance, this study found that questions as a type of engagement strategy were absent from the Arabic corpus of editorials and this finding calls for more attention to the training of editorialists to take advantage of these strategies in their argumentative texts.

## Footnotes

1 We subscribe to Hyland’s (2019a) assertion that “[i]n looking at metadiscourse as functional we can see that metadiscourse is something that we do, a social act through which people carry on a discourse about their own discourse for particular rhetorical purposes” (p. 29). The analysis is functional because metadiscourse markers, of which engagement is one, are non-propositional. As Crismore et al. (1993, p. 40) argued, metadiscourse “does not add anything to the propositional content but that is intended to help the listener or reader organise, interpret and evaluate the information given.”

2 The taxonomy is presented in the Theoretical Framework section.
